# Predictive sustainability in agriculture: Machine learning analysis of active ingredient restrictions and bans

**DOI:** 10.1371/journal.pgph.0004446

**Published:** 2025-04-22

**Authors:** Rodrigo Garcia Brunini

**Affiliations:** University of São Paulo, Rua Quintino Bocaiuva, São Paulo, Brazil; PLOS: Public Library of Science, UNITED STATES OF AMERICA

## Abstract

Developing active ingredients for the global market requires substantial investment, often exceeding 300 million euros. This process takes an average of 12 years from initiation to commercialization. Despite this lengthy timeline, the industry frequently encounters significant restrictions and bans on active ingredients due to stringent international regulations and evolving environmental safety requirements. In this context, the analysis of regulatory lists using advanced machine learning and statistical modeling techniques becomes crucial for identifying the key parameters that influence the restriction and banning of active ingredients. This study aims to provide insights that enhance decision-making processes, thereby contributing to sustainability by reducing unnecessary environmental research and development efforts. The findings indicate that Governmental and Non-Governmental Organizations, as well as Blacklists, are key influencers in the restriction and ban of active ingredients for agricultural use, with *Codex Alimentarius* acting as a regional influencer depending on the specific country. Ultimately, the insights derived from this research can assist industries and policymakers in developing more effective regulatory strategies, promoting sustainable practices, and ensuring that new active ingredients are selected based on comprehensive and informed criteria that consider both safety and environmental impact.

## Introduction

Active ingredients are fundamental molecules for the development of products or processes within the chemical industry, being considered essential elements in the performance of specific chemical functions, whether in food, pharmaceuticals, fuels, hygiene and cleaning products, cosmetics, or even phytosanitary products [1].

It is estimated that the cost for chemical industries to develop a new active ingredient for entry into the global market can exceed 300 million euros. These expenses encompass everything from the initial processes of research and development of the molecule and toxicological studies to the costs of regulations and final product registration. On average, it takes about 12 years from the beginning of the active ingredient’s development to its first sale as a formulated final product [2].

Although the use of active ingredients in formulated products is currently present in virtually all industrial production chains around the globe, some of these ingredients can be considered harmful to health and the environment. As a result, there are strict regulations and standards that vary depending on the laws and norms of a country’s health and food safety agencies, as well as international agreements or specific industry segments. For example, in the case of active ingredients directed towards agriculture, notable references include the Rotterdam Convention (1998) [3] and the Stockholm Convention – 2001 [4], the European Food Safety Authority (EFSA) - 2018 [5], the World Health Organization (WHO) - 2019 [6], and the *Codex Alimentarius* of the Food and Agriculture Organization of the United Nations (FAO) – 2013 [7].

These international organizations establish toxicity level classifications for an active ingredient when used in nature, including acceptable daily intake (ADI), acute reference dose (ARfD), environmental toxicity for mammals, fish, pollinators, and non-target species, among others [8, 9]. These documents are based on robust scientific data focusing on human and environmental health and often compile a “blacklist” of active ingredients considered restricted in specific situations or even prohibited for use in nature.

In the case of the *Codex Alimentarius* managed by the FAO/WHO: Food Standards Programme, acceptable maximum residue limits (MRLs) for an active ingredient are established for certain plant species, such as commodities (corn, soybeans, coffee, cotton, sugar, etc.), or processed foods [10, 11]. These limitations are important as they aim to protect farmworkers, end consumers, and the environment from potential acute and chronic exposures caused by the toxicity of the molecules.

It is important to note that exceeding the maximum residue limit (MRL) in a product of plant or animal origin means that, in practice, the use of the active ingredient has surpassed the recommended safe dose values stated in the labels of the technical or formulated products marketed by the industry [12].

In addition to the official global monitoring and food safety agencies, there are also non-governmental organizations (NGOs) certifying bodies, and global institutions that have the potential to influence authorities and consumers through the dissemination of reports and news related to the monitoring of risks posed by chemical substances used in agriculture [13]. Some of these organizations have a significant direct impact on the global food market, such as ALDI, Utz, Rainforest, among others [14–16]. Thousands of farmers around the world use the protocols of these certifying bodies and NGOs to obtain good agricultural practice certifications, facilitating the entry of their products into national and international markets. This, in turn, directly influences the market for active ingredients specifically used for the maintenance and protection of crops against pests and agricultural diseases [17].

This combination of direct and indirect factors, involving regulations from EFSA, FAO, FDA and WHO, established toxicity levels, maximum residue limits, certifying bodies, and even the influence of NGOs, exerts significant pressure for the restriction and ban of molecules [18–21]. This, in turn, directly impacts the chemical sector and even the food safety of the global population [22].

Only in Brazil, the practical implications of an unexpected restriction or ban by national or international agencies on one of the ten most commercially traded active ingredients in agriculture could directly affect over 78 million hectares of cultivated grain land and compromise approximately 322 million tons of food for human and animal consumption [23, 24].

In this context, analyzing the variables using mathematical machine learning models that have the potential to trigger restrictions or bans on active ingredients aimed at crop protection in the global market is of utmost importance. This analysis can help determine the main influencers behind these banning events and also assist decision-making for leaders in the agricultural chemical industry, enabling them to make more accurate and secure choices for future active ingredients in their portfolios. Furthermore, this analysis has a direct impact on the planet’s sustainability, as it helps prevent unnecessary environmental research involving chemical products and reduces the future impact of a potential molecule that may have a low “shelf life” in the global market. Thus, the objective of this work was to determine the key parameters with the potential to influence the restriction and ban of active ingredients in the global market for agricultural use.

## Materials and methods

To obtain and extract the information, data from open databases, manuals, and reports from the main official food safety and chemical substance control agencies worldwide were used, as well as information from non-governmental organizations and major retail brands that have significant influence in the food market in the main analyzed global regions, as shown in Table 1.

**Table 1:** Main organizations and institutions used as data collection sources.

Organization/Institution	Type
Albrecht-Discount – ALDI [25]	Retailer
JUMBO [25]	
REWE Group [25]	
Agência Nacional de Vigilância Sanitária – Anvisa [8]	National Regulation
Japan Organic Production [25]	
Ministério da Agricultura, Pecuária e Abastecimento – MAPA [26]	
Servicio Nacional de Sanidad y Calidad Agroalimentaria – SENASA [27]	
United States Department of Agriculture – USDA [28]	
CLP-ECHA (Acute toxicity) [25]	Multilateral Regulation
European Food Safety Authority – EFSA [18]	
Globally Harmonized System of Classification and Labelling of Chemicals – GHS [6]	
Joint Meeting on Pesticide Management – JMPM [25]	
Montreal Ozone Depletion [29]	
Pesticide Analytical Manual – PAM [19]	
Rotterdam Convention [30]	
Stockholm Convention on Persistent Organic Pollutants – POP [4]	
The European and Mediterranean Plant Protection Organization – EPPO [31]	
Candidates for substitution (European Comission) [32]	International Regulation
Low-risk substances (European Comission) [32]	
Organic production (European Comission) [32]	
Food and Agriculture Organization of the United Nations – FAO	
Pesticide Environmental Stewardship – PES [33]	
World Health Organization – WHO [34]	
Fairtrade International [14]	Non-Governmental Organization
Global, Good Agricultural Practices - GLOBALG.A.P. [35]	
Minor Use Foundation – MUF [36]	
More Profitable Sustainable – MPS [37]	
PlanetProof [38]	
Rainforest Alliance [15]	
Utz Certified [16]	

For the collection of technical information regarding active ingredients, databases dedicated to agricultural pesticide molecules were utilized, including “The Global Plant Protection Products Database and Maximum Residue Limits (MRLs) – Homologa v.6”, the “PubChem Database” from the National Center for Biotechnology Information in the United States, and the “Pesticide Properties Database (PPDB)”, from the University of Hertfordshire, England [9,25,39].

The variables used to evaluate the influence on restricted and banned active ingredients were based on the characteristics of each active ingredient: Class (Biologicals, Fungicides, Herbicides, and Insecticides), Active Group, Mode of Action, Country of Use, Crop Used, Crop Group, Purpose of Use, Date of Use Update, Expiration Date of Use, and Current Use Status. Additionally, the situation of each molecule was assessed according to the “blacklist” of each organization observed in Table 1, considering government, non-government, and retail entities (taking into account the variables: active ingredient, certification, classification, country, and crop used). Finally, the acceptable maximum residue limits (MRLs) (mg of active ingredient/kg of crop mass) for each active ingredient were obtained through the Codex Alimentarius, based on the active ingredient, country, and crop used. [40].

The justification for using these characteristics in the research is that the agricultural chemical products industry spends a significant amount of resources and labor hours on regulatory issues, discussions about restrictions, residue limits, technical support for producers in the field, and risk analyses. These attributes of each molecule and the variables involved are connected in some way to these aspects.. In addition, a round of discussions was conducted with strategic managers of crop protection products from a multinational company to understand the main risk factors considered by these leaders when selecting an active ingredient for crop protection.

After the extraction of qualitative and quantitative data, the qualitative categorical data on the classification of active ingredients according to the toxicity and restriction “blacklists” were transformed into quantitative data and grouped into impact categories (Non-Governmental Organizations, Government Organizations, and Codex). This grouping was based on the “weight” of the classification; for example, active ingredients with higher toxicity will receive more points (ranging from 0 to 1) than those considered less toxic. In total, more than five million data points were collected and analyzed, organized into a data frame with twenty-two columns as described in Table 2.

**Table 2:** List of qualitative and quantitative indicators of the data frame created for data analysis.

Index	Description
CAS Number	Unique identifier for the chemical substance.
Active Ingredient	Name of the active ingredient/molecule.
Class	Classification of the active ingredient based on its target.
Action Group	Broader category of the pesticide’s mode of action.
Active Ingredient Group	Specific group of the pesticide’s mode of action.
Mode of Action	Mechanism by which the pesticide kills or controls pests.
Country	Location where the registration, restriction, or prohibition of use occurs.
Continent	Regional grouping for the data of the active ingredients.
Crop Group	Broad category of crops for which the molecule is or is not registered for use.
Crop	Specific crop according to the molecule.
Active Ingredient Status	Regulatory status of the active ingredient for the crop.
Last Update	Most recent update of the active ingredient registration in the country.
Expiration Date	Expiration or authorization of the active ingredient.
MLR (ppm)	Maximum residue limit in (mg/kg) for the active ingredient.
Restriction or Ban Status	Regulatory status of the active ingredient for the crop according to the country.
Certification	Certification required for the use of the pesticide product.
Classification	Classification of the active ingredient based on its hazard level.
Organization	Organization that registered or authorized the molecule.
Impact of Non-Governmental Organizations (Blacklist)	Impact calculation for the active ingredient based on criteria from NGOs, Retailers, and Certifiers, and country regulation.
Codex (Impact of *Codex Alimentarium)*	Impact calculation for the active ingredient according to the crop and established Maximum Residue Limits (MRLs).
Organizations (Impact of Government)	Impact calculation for the active ingredient according to the regulatory status of governmental organizations (International and Multilateral).
Total Impact (Total Impact on Restrictions and Bans)	Overall numerical representation of the pesticide’s impact (Blacklist + Organizations + Codex).

Analyzing more than twelve hundred active ingredients, distributed across thirty countries covering all continents (except Antarctica) and three hundred crops for agricultural purposes.

The positioning of the Codex as a separate group is justified because, for this document, the residue limits for pesticides are specifically applied according to the class of molecules observed and the agricultural crop, requiring different weights based on the value of the established maximum residue limit (MRL) (the more restrictive, the higher the impact weight). This differentiates it from other lists, both governmental and non-governmental, which tend to be more general in their positioning on the restriction or banning of an active ingredient, using terms such as: banned, expired, pending, or restricted. In this sense, values assigned by the Codex were established, with weights designated for calculating the impact in each observed group varying from 0 (no restrictions and MRLs) to 1 (maximum restriction with MRLs equal to 0.01 mg/kg – the highest restriction limit).

In order to more effectively observe the global influence of data from multilateral and national organizations, countries were allocated according to geopolitical and geographical regions [41].

To analyze the relationship between the influencing variables (Restrictions, Governmental Organizations, Codex, and Non-Governmental Organizations) and to measure the strength and direction of the linear relationship between these pairs of variables regarding the impact on the banning of active ingredients, Pearson’s correlation (1909) was employed for each class of active ingredient [42].

This is a statistical measure that evaluates the linear relationship between two continuous variables. It ranges from -1 to 1, where values close to 1 indicate a strong positive correlation, values close to -1 indicate a strong negative correlation, and values close to 0 indicate a weak or nonexistent correlation.

Pearson’s correlation is widely used in statistics and data analysis to identify relationships between variables, and it can be useful in determining which organizations (Governmental, Non-Governmental, and Codex) used in this study have a stronger and more significant relationship with restrictions and bans according to the class of active ingredient (Biologicals, Insecticides, Herbicides, and Fungicides). This understanding is crucial for recognizing which bureaucratic and hierarchical compositions are more likely to affect the availability of these products in the market.

Multiple Correspondence Analysis (MCA) was applied, a statistical technique developed by Fischer (1940) that allows for the analysis of relationships between multiple categorical variables. This method is useful for identifying patterns and associations among categorical variables, which can be challenging to detect with other statistical techniques, particularly in observing the influence of the other categorical variables used in this study (Table 2) on the restrictions and bans of active ingredients.

The advantage of MCA is that it enables the visualization of associations between the studied categories, allowing for the construction of “theoretical zones” and the production of results whose effectiveness lies in revealing connections [43].

The interpretation of an MCA graph involves analyzing the position of the points on the graph, which represent categories of variables. The distance between the points indicates the similarity between the categories, while the direction of the points may indicate positive or negative relationships. The graph can also be divided into quadrants, which can be used to interpret the relationships between the categories. By considering the characteristics of the variables and the relationships among them, it is possible to interpret the MCA graph and gain valuable insights into the relationships between the analyzed variables [43].

In the context of the study on the restriction and banning of active ingredients in the global market for agricultural use, MCA can be useful for identifying which categorical variables, such as type of active ingredient, country, type of crop, active group, class, and other analyzed variables, have a stronger and more significant relationship with restrictions and bans. This is achieved by identifying patterns and associations among these variables according to each class of chemical product.

The multinomial logistic model proposed by Cox (1958) was applied, which is a statistical technique used to model the probability of a dependent categorical variable taking on a specific value based on a set of independent variables. This model is an extension of the binary logistic model, allowing for the analysis of categorical variables with more than two levels. The multinomial logistic model is particularly useful when analyzing the relationship between a dependent categorical variable and a set of independent variables, as well as when predicting the probability of a particular category being chosen [44].

In the study on the restriction and banning of active ingredients in the global market for agricultural chemical products, the multinomial logistic model can be used to analyze the relationship between independent variables (such as type of active ingredient, MRL, toxicity, active group, type of crop, etc.) and the probability of restriction or banning. Furthermore, the model allows for predicting the probability of restriction or banning for different combinations of independent variables and identifying which influencers have greater or lesser contributions to the banning or restriction process. This enables a robust study of the causes that most impact regulatory decisions, providing a broad direction on regulatory influence variables according to the different classes of active ingredients..

For data collection, standardization, statistical analysis, and machine learning, the Spyder software [45] was used, which is a development environment for the Python programming. Additionally, specific libraries for data analysis and machine learning were utilized, including Pandas for data manipulation and analysis, Geopandas for graph creation and visualization and NumPy for mathematical operations [46], Matplotlib and Seaborn for data visualization [47, 48], Plotly for interactive data visualization [49], Scikit-learn for machine learning, including the implementation of Random Forest [50], and Statsmodels [51], for statistical analysis. The combination of these libraries allowed for a comprehensive and robust analysis of the data, facilitating the attainment of accurate and reliable results. [46,52].

## Results and discussion

According to the qualitative analysis, the active ingredient with the highest frequency is Pyrethrin (or pyrethrin extract), which, despite being a plant-derived insecticide, has high toxicity for bees and non-target insects [9]. This fact may explain the classification of this molecule in the analyzed data frame, as toxicity to pollinators is one of the factors most involved in the processes of restrictions and bans on pesticides in agriculture [53].

**Fig 1:**
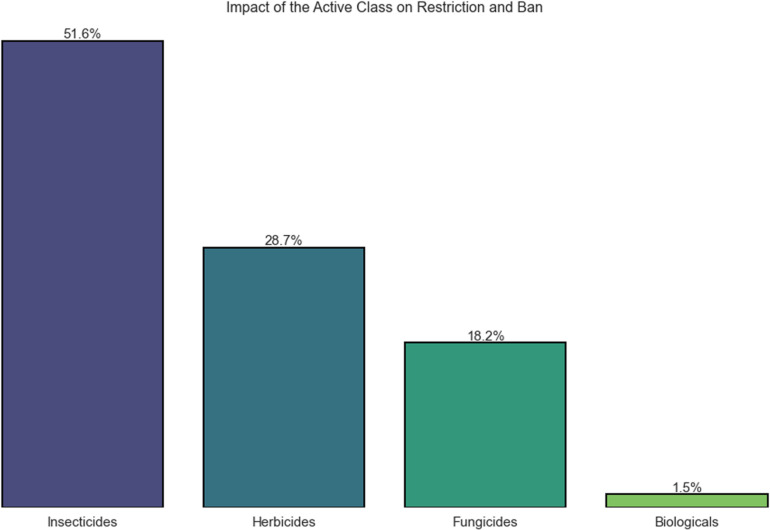
Impact of pesticide classes regarding restrictions and bans on active ingredients for agricultural use.

In Fig 1, the importance of the active ingredient class in cases of banning is observed, with insecticides exceeding 50% compared to other classes. This is directly related to the requirements of regulatory agencies for active substances with high potential environmental risks. [54] emphasize the importance of a wide range of studies involving pollinators, non-target insects, and animals in the decision-making process for the registration of a new pesticide. In the case of insecticides, the potential for restriction and banning increases due to their high demand for studies involving environmental risk assessments, as can be observed in the graph, where the Biological class accounts for only 1.5% of the restrictions and bans.

**Fig 2:**
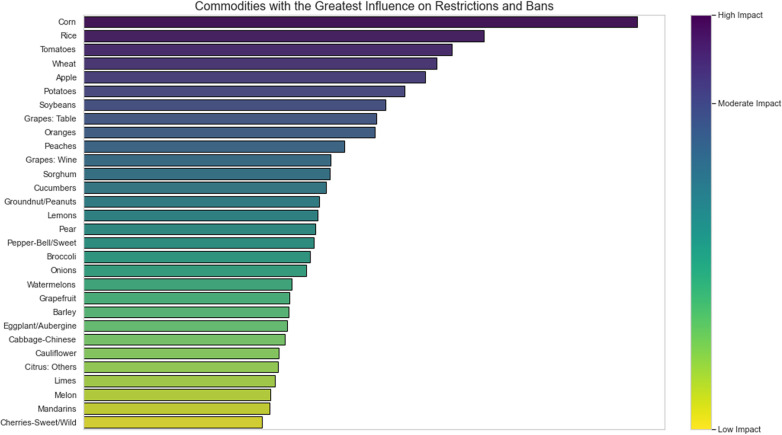
The crops with the greatest influence on the impact regarding restrictions and bans on active ingredients for agricultural use.

In Fig 2, the analyzed agricultural crops are highlighted, where corn (*Zea mays*), rice (*Oryza sativa*), tomato (*Solanum lycopersicum*), and wheat (*Triticum spp*.) are identified as the commodities with the greatest influence on the impact of restrictions and bans on active ingredients.

In addition to the fact that these crops have undergone long domestication processes by humans over the ages and have established themselves in practically all territories of the globe, they are commodities used directly for human consumption. This suggests extensive use of plant protection products aimed at these crops, leading to greater restrictions and bans [55]. Also noteworthy is soy, which is one of the commodities with the highest exports among countries, with a large portion of its produced volume being allocated for animal feed.

**Fig 3:**
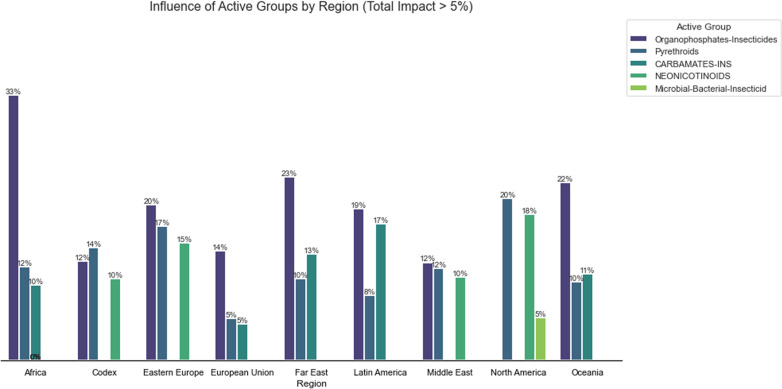
Groups of active ingredients with the greatest influence on restrictions and bans for the studied regions.

In Fig 3, the influence of active groups on the total impact (> 5%) is shown, along with the distribution of these actives for one of the analyzed regions, including the *Codex Alimentarium*, as it is an important reference used by various countries worldwide regarding the permissible residue limits in crops. The importance of restrictions and bans on Organophosphates, Pyrethroids, Carbamates, and Neonicotinoids is evident, as all of these are chemical groups within the class of insecticides [56].

An interesting data point observed is the representation in North America for some microbiological active ingredients that control bacteria and insects. Even though these are products with biological active principles, there are cases involving regional regulations on restrictions and bans for this class of active ingredients.

**Fig 4:**
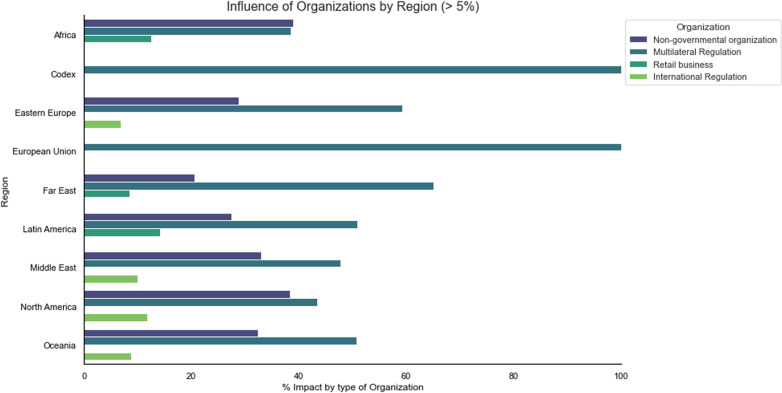
Influence on the impact of restrictions and bans by organizations for the major regions of the globe.

According to the data in Fig 4, regarding the influence of restrictions and bans above 5% from organizations around the globe, it is observed that groups with multilateral regulations have a greater influence in almost all regions and the *Codex*, particularly in the European Union and the Far East, followed by non-governmental organizations and retailers, which have significant commercial influence in Latin America. This fact can be understood due to the economic policies regarding commodities adopted by European and Asian countries, which are among the top international importers of agricultural products from emerging countries such as Brazil, Argentina, Colombia, and others [57].

**Fig 5:**
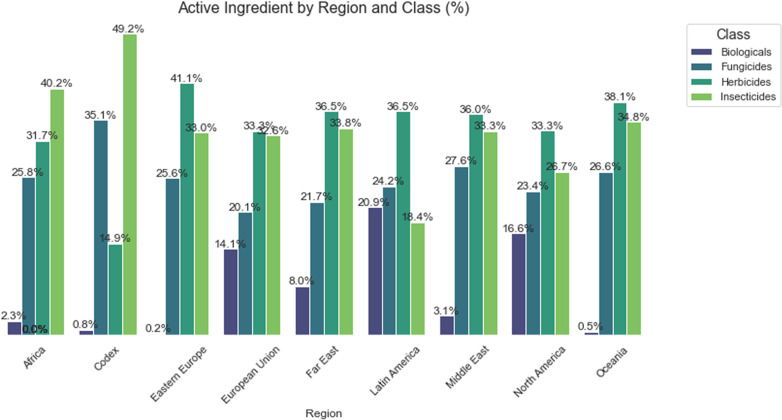
Percentage distribution of registered active classes for the major regions of the globe.

For Fig 5, it is shown that the classes of herbicides and insecticides predominate in the regions of the European Union, Oceania, and Latin America, representing about 40% to 50% of the registrations. This may be associated with the high demand for commodities in these areas and, consequently, the prevalence of pests and diseases in agricultural lands [58].

Herbicides hold a significant position in East Asia, the Middle East, and North America, which may be associated with the type of agriculture and farming techniques used in agricultural production in these regions. This data can also be observed according to the FAO, which conducted a two-decade survey of the use of active ingredients by the main commodity-exporting countries globally [20].

According to Fig 5, the class of Biologicals shows an interesting participation in the European Union (14%) and Oceania (8%), suggesting a potential for growth of sustainable products, considering regions where there is greater regulatory pressure on the registration of active ingredients for agricultural use.

The regionalization of restrictions and bans on active ingredients intended for agriculture is an important factor in understanding how regulations, certifications, and the commodities market have a direct influence on the use of pesticides. In this sense, the data in Table 3 highlight how complex and diverse this influence is across global regions.

**Table 3:** Influence of geographic regions on the restriction and ban of agricultural active ingredients.

Region	Total Influence on Impact
Asia	32.2%
North America	26.3%
European Union	13.8%
Middle East	10.1%
South America	7.2%
Oceania	6.8%
Africa	3.6%
Eastern Europe	2.4%

It is observed that Asia is the most influential region in the restrictions and bans of active ingredients for agricultural use (32.2%). This value may be linked to the fact that the region is one of the largest importers of grains and other global commodities, while also having a larger number of countries than the other analyzed regions. Many of these are major agricultural producers that adopt stringent regulations in response to growing concerns about food and environmental safety [59]. North America and Europe follow with 26.3% and 13.8% influence, respectively, reflecting their historically cautious approaches toward pesticides, particularly concerning the concentration of multilateral regulatory agencies in these regions, such as the EFSA and the USDA.

**Fig 6:**
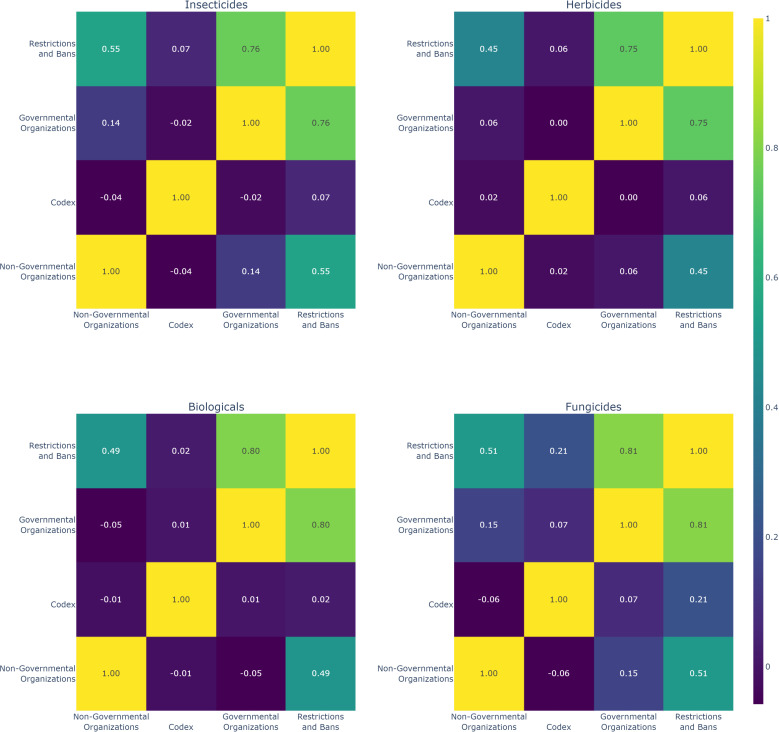
Pearson correlation applied to each class of active ingredients: insecticides, herbicides, fungicides, and biologicals, for the variables influencing banning.

According to the Pearson correlation data, for each class of active ingredient (Fig 6), insecticides, herbicides, fungicides, and biologicals show a strong influence of governmental organizations on restrictions, with correlations of 0.76, 0.75, 0.81, and 0.80, respectively. Non-governmental organizations also show significant correlations, ranging from 0.45 to 0.55, indicating their relevant contribution, although it is lower than that of governmental entities. The *Codex Alimentarius* demonstrates weak correlations, suggesting a less direct influence on regulatory decisions.

These data suggest that the high correlation values between governmental organizations and restrictive measures against active ingredients reflect the effects of regulatory policies, predominantly guided by national or international legislations and authorities.

It is well known that these organizations play a monopolistic role in the risk assessment associated with pesticides, justifying their strong influence on the banning of active ingredients [60]. The correlation with NGOs illustrates the increasing pressure exerted by these groups on environmental protection and public health, although, as observed, their influence is less significant than that of governments.

The weak relationship of the Codex with the other variables and classes (Fig 6) may be linked to its role as a global reference influencer in restriction and banning policies for various countries. Thus, higher correlations among regulatory bodies highlight the significance of their influence on restriction policies, while the Codex appears to play a supplementary role in this action.

**Fig 7:**
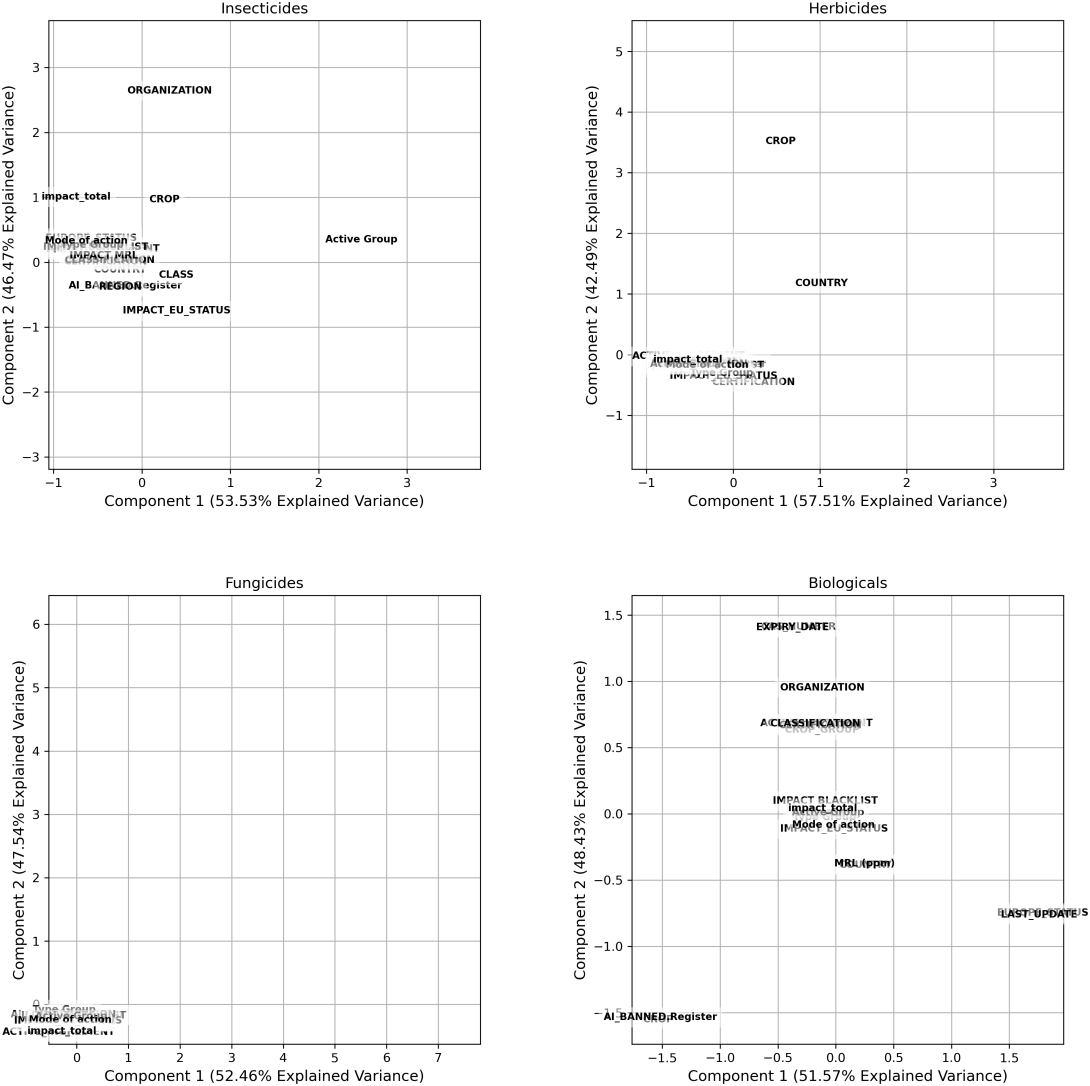
Multiple Correspondence Analysis by Fischer, applied to each class of active ingredients, including insecticides, herbicides, fungicides, and biologicals, for the influencing variables on banning.

The Multiple Correspondence Analysis (MCA) for different classes of active ingredients (Fig 7) highlights the influence of regulatory variables, countries, regions, crops, crop groups, active ingredients, type of active ingredient, and mode of action on the restrictions of each class.

For insecticides (Fig 7), components 1 and 2 explain 52.46% and 47.54% of the variance, with a strong association between non-governmental organizations, restrictions, the type of active ingredient, the crop group, and the region. For herbicides, the variances are 54.75% and 45.25%, indicating that the variables, pressure from NGOs and governmental organizations, *Codex*, and characteristics of the active ingredients have a greater relationship with each other. For fungicides, the variances were 53.64% and 46.36%, demonstrating a stronger relationship between NGOs, countries, Codex, and attributes related to the characteristics of the active ingredient. Meanwhile, for biologicals, the data indicate variances of 54.78% and 45.22%, showing greater separation among the variables, with closer proximity to factors such as region, NGOs, and the mode of action of the active ingredients.

The ACM graphs in Fig 7 indicate that the characteristics of a restriction or ban are shaped by the connection between regulatory, social, and associated variables. The strong relationship between governmental organizations and restrictions suggests that decision-making is based on the risk assessments conducted by these bodies. Meanwhile, the pressure from NGOs and international standards reflects an increase in the adoption of sustainable agricultural practices. This demonstrates that, despite the differences among pesticide classes, the dynamics between regulations and external pressures are a common factor shaping restriction policies. Consequently, this also establishes that laws and standards are influenced not only by public health concerns but also by regional and environmental aspects in the use of active ingredients targeted for agriculture [61].

The Multiple Correspondence Analysis (MCA) supports the importance of this type of analysis in affirming that influencing agents vary for each class of active ingredient, and each class should be treated independently regarding aspects of restrictions and bans.

**Fig 8:**
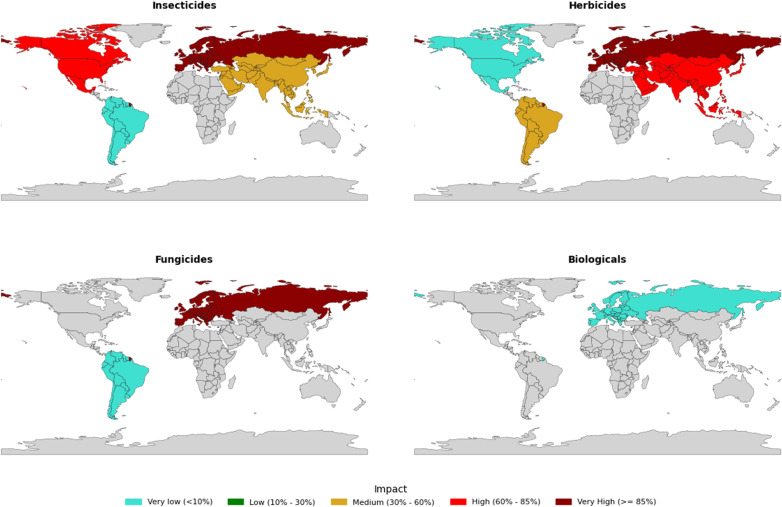
Maps of global regions with the greatest influence on the banning of insecticides, herbicides, fungicides, and biologicals. Created using the open source library: Natural Earth, available in the documentation: https://www.naturalearthdata.com/downloads/10m-cultural-vectors/10m-admin-0-countries/.

In Fig 8, it can be observed that the global influence maps on the banning of insecticides, herbicides, fungicides, and biologicals exhibit significant regional differences related to the analyzed variables that exert regulatory force over the use of these active ingredients.

In general, the European continent shows the greatest influence on the impact of active ingredients for insecticides, herbicides, and fungicides, with very high values represented in dark red (above 85%), while also exhibiting very low influence for biologicals (≤ 10%). Asia, on the other hand, exerts greater influence on insecticides and herbicides, with high values in red (between 60% and 85%), reflecting the increasingly stringent regulatory positioning of these regions (Europe, Russia, and China), which are major global importers of commodities. These regions are implementing stricter policies regarding the use of these elements in domestic agriculture, while also applying pressure on the importation of food from emerging countries, particularly those in South America [59].

In the case of the Americas (North and South), the influence is generally low to moderate (Fig 8) for insecticides, herbicides, and fungicides. This can be attributed to the fact that these strategic regions are primarily responsible for ensuring food security for populations by supplying thousands of countries worldwide. From the perspective of the agribusiness sector on a global scale, it would not be advantageous for these countries to implement overly strict restrictive policies on inputs that are essential for maintaining productivity and delivering commodities to the rest of the world.

This situation can be seen as ironic in light of the economic and sustainable development goals outlined by the UN Agenda for 2030 [62]. Regions that are economically dependent on agriculture, such as countries in Africa, Australia, Argentina, Brazil, Colombia, and others, are the ones most affected by pressures from regulations, certifications, and rules concerning the restriction and banning of active ingredients. This contrasts with regions that have a higher demand for commodities, which impose bureaucratic measures that adversely affect agricultural work and industry while also demanding lower prices for commodity exports.

Given the high demands for active ingredients that employ increasingly efficient technologies and promote sustainability, the decision-making process within industries—whether to continue researching a new agricultural active ingredient or to cancel projects likely to face future restrictions and bans—is crucial. This approach is essential for mitigating economic losses and fostering predictive sustainability.

In this context, the use of binary and multinomial models [44] is justified for predicting potential restrictions and bans on active ingredients influenced by the most relevant observed variables: Organizations, Blacklist, and Codex, for each class, as shown in Table 4.

**Table 4:** Results obtained from logistic regression for each class of active ingredient: insecticides, fungicides, herbicides, biologicals, and all classes combined.

Class	Tr^(1)^	It^(2)^	Ac^(3)^	Sb^(4)^	Ep^(5)^	Pr^(6)^	LL^(7)^	CI^(8)^	CB^(9)^	CO^(10)^	CC^(11)^
All	$5.18^{6}$	7	0.66	0.88	0.65	0.31	$-2.49^{10}$	-2.06	-2.22	3.19	2.13
Ins	$2.51^{6}$	7	0.61	0.86	0.60	0.23	$-1.33^{10}$	-1.55	-2.09	2.56	1.91
Her	$9.87^{5}$	6	0.63	0.89	0.62	0.27	$-5.02^{9}$	-1.88	-2.14	2.90	1.31
Fun	$9.53^{5}$	8	0.74	0.95	0.74	0.47	$-3.51^{9}$	-2.31	-5.76	3.59	2.35
Bio	$7.28^{5}$	35*	0.88	1.00	0.88	0.72	$-1.41^{9}$	-41.1	-62.9	43.1	-22.9

Note: ^(1)^ Training [Tr]; ^(2)^ Iterations [It]; ^(3)^ Accuracy [Ac]; ^(4)^ Sensitivity [Sb]; ^(5)^ Specificity [Ep]; ^(6)^ Pseudo R^2^ [Pr]; ^(7)^ Log-Likelihood [LL]; ^(8)^ Coef. Intercept [CI]; ^(9)^ Coef. Blacklist [CB]; ^(10)^ Coef. Organizations [CO]; ^(11)^ Coef. *Codex* [CC].

* did not converge.

The data in Table 4 reveal the results of the logistic regression for each class of active ingredient regarding restrictions and bans, where the influencing variables are the weight of the Blacklist, Organizations (NGOs and Governments), and the *Codex*. For active ingredients classified as insecticides, there is a significant negative impact from the Blacklist variable (coef. -2.0937), contrasting with the positive influence of Organizations (coef. 2.5634) and the *Codex* (coef. 1.9179), both of which increase the likelihood of restrictions and bans. However, the accuracy and specificity achieved values of 0.61 and 0.60, respectively, indicating that the prediction model for restrictions and bans demonstrates moderate potential in forecasting for the insecticide class (Equation 1).


$$P\left(Res\_Ban|Insecticides\right)=\frac{1}{1+e^{-\left(-1.5502-2.0937*Blacklist+2.5634*Organizações+1.9179*Codex\right)}}$$ (1)

For herbicides, the results found values for the Blacklist (coef. -2.1423) and Organizations (coef. 2.9066) that were quite similar to those of other classes. However, the Codex variable (coef. 1.3189) showed a lesser effect on the model (Equation 2). The accuracy, sensitivity, and specificity data indicated values of 0.63, 0.89, and 0.62, respectively, also suggesting a reasonable capacity for predicting restrictions and bans.


$$P\left(Res\_Ban|Herbicides\right)=\frac{1}{1+e^{-\left(-1.8865-2.1423*Blacklist+2.9066*Organizações+1.3189*Codex\right)}}$$ (2)

In the case of fungicides, the model achieved better performance with a robust fit (Pseudo R²: 0.4699), showing a significant effect in Equation 3 from the variables Blacklist (coef. -5.7626), Organizations (coef. 3.5998), and Codex (coef. 2.3512), indicating a greater influence on restrictions and bans for this class. Regarding effectiveness, sensitivity, and accuracy (0.74, 0.95, and 0.74, respectively), the model demonstrated greater predictive potential compared to the others.


$$P\left(Res\_Ban|Fungicides\right)=\frac{1}{1+e^{-\left(-2.3114-5.7626*Blacklist+3.5998*Organizações+2.3512*Codex\right)}}$$ (3)

However, for the biological class, there were difficulties in establishing an effective model, as attempts showed imprecision in converging, indicating adversity related to the near-complete isolation of the data. Even with high accuracy and sensitivity values (0.88 and 1.00, respectively), the high interaction values (35) indicated overly burdensome results and low robustness in the model, suggesting that these variables alone are not sufficient to effectively explain the restrictions and bans. This is not surprising in practice, as organizations, retailers, certifiers, and other stakeholders highlight the class of biologicals as a viable alternative for replacing chemical active ingredients [63].

When comparing the global model for all classes of active ingredients (Equation 4), it shows functional performance according to the variables Blacklist (coef. -2.2260), Organizations (coef. 3.1949), and *Codex* (coef. 2.1397), which maintained applicable influence, despite moderate values for specificity, accuracy, and sensitivity categories (0.66, 0.65, and 0.88, respectively).


$$P\left(Res\_Ban|AllClasses\right)=\frac{1}{1+e^{-\left(-2.0630-2.2260*Blacklist+3.1949*Organizações+2.1397*Codex\right)}}$$ (4)

**Fig 9:**
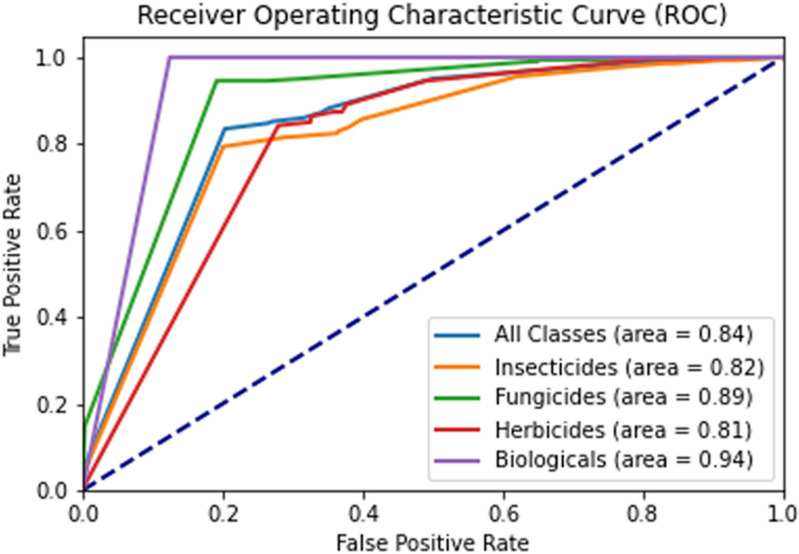
ROC curve to discriminate the cases of influence on restrictions and bans for the models of different classes of active ingredients.

To visualize and compare the adherence of the logistic prediction models for restrictions and bans, a Receiver Operating Characteristic (ROC) curve analysis was conducted for each class of active ingredients and also for all classes, as shown in Fig 9.

According to the areas of the logistic models under the curve (AUC) as dotted, it is possible to observe the classification ability of the models for each curve of the classes of active ingredients, with values closer to 1 indicating better performance [64]. However, as previously noted, even though the logistic model for biologicals achieved the largest area under the curve (0.94), it does not indicate efficiency in distinguishing between bans and non-bans.

For the areas under the curve of the logistic models for fungicides, insecticides, and herbicides, the values were 0.89, 0.82, and 0.81, respectively, demonstrating high and moderate performance, as shown in Table 3. In the case of the global model for all classes, the area under the curve was 0.84, indicating a balanced potential when compared to the other curves. This demonstrates the possibility of using these logistic prediction models as a practical tool to assist industries and leaders in making better decisions regarding the influencers that most impact the restrictions and bans on active ingredients.

These results confirm that the statistical analyses and models used are essential for identifying the key relevant variables in cases of restrictions and bans on active ingredients used in agriculture.

The interrelationship between cases of bans and restrictions, along with regulatory, bureaucratic, and regional characteristics, has proven to be particularly important. The pressures exerted by the influencers highlighted in this research have the potential to determine the fate and “shelf life” of active ingredients intended for agriculture.

Consequently, identifying influencers and their connection with laws regarding the use of active ingredients in the agricultural sector is crucial for risk analysis by corporations. This scrutiny calls into question the future of agribusiness in various emerging countries and creates a new competitive niche that we can elucidate as “predictive sustainability.” In this context, the decision is made to ensure no negative impact on the environment, based on the observed variables and their direct influence on business practices.

Companies that can adopt a business intelligence approach promoting innovative, data-driven tools that enable quick responses to regulatory pressures and the growing demand for environmentally friendly products are destined to be a “success case” in the global market, while also saving billions of dollars.

Thus, this research not only highlights the key influencers in the restriction and ban of active ingredients for agriculture but also presents a critical view of how these variables and their stakeholders direct a more sustainable future solely for a select group of decision-making countries. This imbalance affects productivity, food security, and environmental impact for agricultural commodity-exporting countries.

## Conclusions

The main parameters influencing the restriction and banning of active ingredients for agricultural use are governmental and non-governmental organizations, as well as blacklists. These organizations employ restrictive policies based on data regarding environmental toxicity, social cancellation pressures, and agricultural certification protocols that shape the commodities market, respectively.

The *Codex Alimentarius* can be considered a regional influencer depending on the usage by the requesting country.

Different geographical regions diverge in the impact of restrictions and bans according to the class of active ingredients, due to their political and regional particularities regarding issues involving the environment, agriculture, and food security.

It is possible to use statistical models to assist in decision-making in relation to the influencers identified in this research, which have the most impact on corporate leadership decisions in the search for new active ingredients for agricultural use.
